# Mediastinal Fibrosarcoma in a Dog–Case Report

**DOI:** 10.3389/fvets.2022.820956

**Published:** 2022-02-09

**Authors:** Alysha M. McGrath, Sarah A. Salyer, Amanda Seelmann, Alycen P. Lundberg, Melissa R. Leonard, Joshua N. Lorbach, Sarah Lumbrezer-Johnson, Eric T. Hostnik, Giovanni Tremolada, Janis Lapsley, Laura E. Selmic

**Affiliations:** ^1^Department of Veterinary Clinical Sciences, The Ohio State University, Columbus, OH, United States; ^2^Department of Veterinary Clinical Medicine, University of Illinois, Urbana, IL, United States; ^3^Department of Veterinary Biosciences, The Ohio State University, Columbus, OH, United States

**Keywords:** fibrosarcoma, mediastinum, dog, surgery, blunt dissection, chemotherapy, doxorubicin, case report

## Abstract

This represents the first published case report of mediastinal fibrosarcoma in a dog. An 8-year-old male neutered mixed breed dog was presented for evaluation of lethargy and increased panting. Thoracic focused assessment with sonography for trauma revealed moderate pleural effusion. Thoracic radiograph findings were suggestive of a cranial mediastinal mass. Computed tomography revealed a mass within the right ventral aspect of the cranial mediastinum. On surgical exploration, a cranial mediastinal mass with an adhesion to the right cranial lung lobe was identified and removed en-bloc using a vessel sealant device and requiring a partial lung lobectomy. Histopathology results described the cranial mediastinal mass as fibrosarcoma with reactive mesothelial cells identified within the sternal lymph node. The patient was treated with systemic chemotherapy following surgical removal. To date, the dog has survived 223 days following diagnosis with recurrence noted 161 days following diagnosis and radiation therapy was initiated. Primary cranial mediastinal fibrosarcoma while a seemingly rare cause of thoracic pathology in dogs, should be considered in the differential diagnosis for a cranial mediastinal mass.

## Introduction

Mediastinal masses are commonly diagnosed in dogs and cats (1) and can be categorized by anatomic location including craniodorsal, cranioventral, caudodorsal, caudoventral and hilar with cranioventral overrepresented (2). While benign differentials are possible, such as cyst, abscess, granuloma, or hematoma (3), mediastinal tumors located to the cranioventral region occur more commonly with thymoma and lymphoma predominantly represented (4). Sarcomas and ectopic thyroid carcinomas have also been reported though both are considered rare (5).

Fibrosarcoma is defined as a malignant mesenchymal neoplasm caused by an abnormal proliferation of fibroblasts (6). This neoplasm arises most commonly in the skin, subcutaneous tissues and oral cavities of domestic species with internal sites of origination less common (7).

To the authors' knowledge, this is the first report in a dog to describe primary mediastinal fibrosarcoma. The purpose of this case report is to describe the clinical presentation, diagnostic approach, treatment and outcome of fibrosarcoma in the mediastinum of a dog.

## Case Presentation

An 8-year-old male neutered mixed breed dog was presented for assessment at a veterinary practice following a few days of lethargy and increased panting. Thoracic radiographs were performed revealing subjectively moderate pleural effusion. Referral was recommended. The dog presented to the University of Illinois Emergency Service and initial examination revealed tachycardia (170 beats per minute) and tachypnea (50 breaths per minute). Thoracic auscultation revealed muffled heart sounds, decreased lung sounds and a respiratory sinus arrhythmia. The dog was normotensive (144/88 with a mean arterial pressure of 94 mmHg). The remainder of the physical examination was within normal limits. Venous blood gas revealed hyperlactatemia (3.6 mmol/L; reference interval (RI): 0.4–2.93 mmol/L) but was otherwise unremarkable. Thoracic focused assessment with sonography for trauma (TFAST) revealed a moderate amount of fluid in the pleural space bilaterally. Thoracocentesis was performed bilaterally and yielded 1 liter of fluid. The fluid packed cell volume and total protein were 39% and 4.2 g/dl which was consistent with blood as the peripheral packed cell volume and total protein were 45% and 6.5 g/dl, respectively. Cytology of the pleural fluid was consistent with hemorrhagic effusion (nucleated cell count: 15,130 cells/ μl, red blood cell (RBC) count: 5,690,000 cells/μl, total protein: 4.6 g/dl, specific gravity: 1.029 and 100-cell differential count consistent with 56% macrophages, 40% nondegenerate neutrophils and 4% small lymphocytes) with no infectious organisms nor neoplastic cells identified. Given the diagnosis of hemothorax, prothrombin time (PT) and partial thromboplastin time (PTT) were assessed, and findings were relatively unremarkable [PT: 17.5 s (RI: 14.0–19.0 s) and PTT: 128.1 s (RI: 75.0–105.0 s)]. Thoracic radiographs were completed post-thoracocentesis revealing a possible cranial mediastinal mass with residual moderate pleural effusion and mild pneumothorax (suspected to be iatrogenic). Malignant neoplasia was the primary imaging differential. The dog was hospitalized overnight for supportive care and monitoring, which included intravenous (IV) fluids (lactated ringers solution (LRS) at 40 ml/kg/day), aminocaproic acid (50 mg/kg every 6 h) IV and trazodone (3.1 mg/kg as needed every 6 to 8 h) per os (PO) and was transferred to the medical oncology service.

**Figure 1:**
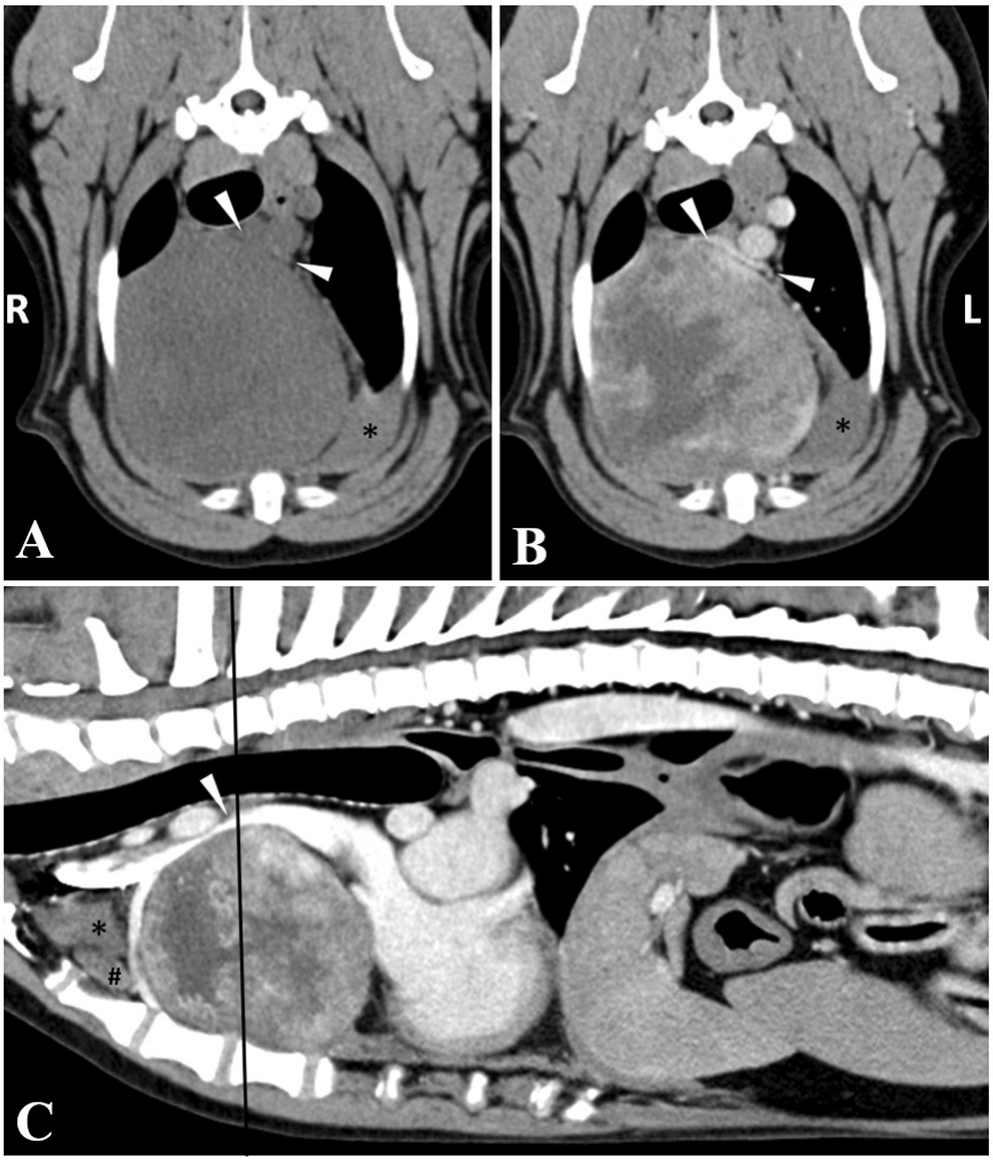
Transverse **(A,B)** and sagittal **(C)** plane CT images of the cranial mediastinal mass with a black line representing the location on the sagittal image. The cranial vena cava (arrowheads) is compressed ventrodorsally by the large mediastinal mass, being bordered dorsally by the brachiocephalic trunk. The mass is soft tissue attenuating, with heterogeneous contrast enhancement, and a well-defined margin. Note the pleural (*) and mediastinal (#) fluid that is not contrast enhancing. Window width and window level were adjusted to 300/40 (soft tissue window), the slice thickness was 2 mm, and the image was acquired with 120 kVp.

The following day, an ultrasound-guided fine needle aspirate of the thoracic mass was performed. While the cytology sample was low in cellularity, mesenchymal cells with moderate cellular atypia were visualized concerning for sarcoma with evidence of hemorrhage. As surgical resection is often recommended for this tumor type, a computed tomography (CT) scan of the thorax and abdomen was performed under sedation (0.2 mg/kg butorphanol IV and 4.0 mcg/kg dexmedetomidine IV), using a multidetector, 128-slice Siemens at 120 kVp and 250 MAs with slice thickness of 2.0 mm and pitch of 0.6. The study was reformatted into dorsal, sagittal and transverse planes using 2.0 mm slice thickness for soft tissue algorithm/display and retro-reconstruction into 1.5 mm slice thickness for lung algorithm/display. Pre- and post-contrast imaging was performed using 60 ml (1 ml/kg) of iohexol administered IV. The CT showed a 9.5 cm x 8.5 cm x 8.7 cm well-defined, round, soft tissue attenuating, heterogeneously contrast-enhancing mass within the right ventral aspect of the cranial mediastinum causing focal compression of the cranial vena cava (Figure 1). Within the mediastinum surrounding the mass, numerous, ill-defined, soft tissue attenuating striations and multifocal pockets of non-contrast enhancing fluid was noted. There was a moderate amount of non-contrast enhancing fluid within the pleural space bilaterally causing retraction and rounding of the lung lobes. Amongst the pleural effusion, there were numerous, small, hyperattenuating (pre- and post-contrast) lobular, broad-based, soft tissue structures along the pleural surfaces. The pleural margin was thickened with contrast enhancement. There were bilateral, ventrally distributed mixed alveolar and unstructured interstitial patterns. There was mild enlargement and rounding of the sternal lymph nodes (1.3 cm in maximum dimension). Given the right-sided nature of the mass, an ectopic thyroid carcinoma was considered the most likely differential diagnosis with thymic epithelial tumor, thymic lymphoma or thymoma considered less likely. The faint hyperattenuating lobular soft tissue along the pleural surface may be due to reactive mesothelium, pleuritis, dependent/stratified blood product, or malignant extension of a cranial mediastinal neoplasia (8). Pulmonary infiltrates within the ventral (dependent) lung immediately adjacent to the increased pleural soft tissue could represent either pulmonary atelectasis due to the space occupying nature of the pleural effusion; alternatively, pulmonary hemorrhage or neoplastic infiltrates. Other findings included mild enlargement and rounding of the right medial iliac lymph node (9 mm in maximum dimension) and an ill-defined, tubular, centrally fluid attenuating and peripherally contrast-enhancing lesion associated with the lateral aspect of the right anal gland (measuring 1.6 cm x 0.8 cm) suspected to be a right-sided anal gland abscess or tail base abscess. It was recommended to proceed with surgical removal of the mass at another institution and submission of the mass for histopathology to guide further treatment. In the meantime, yunnan baiyao (15.6 mg/kg every 12 h) PO was prescribed.

The dog was presented to the Ohio State University Emergency Service the following day. Physical examination was largely unremarkable aside from rectal examination. The anal sacs palpated bilaterally inflamed with purulent material expressed. Point of care bloodwork (blood gas and electrolytes, blood urea nitrogen/creatinine) revealed no significant findings. TFAST revealed a scant to mild amount of pleural fluid bilaterally. The dog was hospitalized overnight for supportive care and monitoring including trazodone (4.8–9.7 mg/kg every 8 h as needed) PO and acepromazine (0.02 mg/kg every 6 h as needed) IV then transferred to the surgical oncology service for surgery. Pre-operative complete blood count and chemistry panel revealed a mild normocytic, hypochromic, non-regenerative anemia (33%; RI: 40.0–55.0%), mild monocytopenia (0.08G/ul; RI: 0.1–1.1G/L), mildly elevated aspartate amino transferase (AST, 77 IU/L; RI: 16–51 IU/L) and mildly elevated total bilirubin (0.28 mg/dl; RI: 0.05–0.2 mg/dl). Clotting factors (PT and PTT) were relatively unremarkable [PT: 16.1 s (RI: 14–19 s) and PTT: 108 s (RI: 75–105 s)]. Thromboelastography revealed no major abnormalities.

The dog was premedicated using midazolam (0.2 mg/kg) IV and methadone (0.2 mg/kg IV), induced using propofol (4.3 mg/kg IV), lidocaine (2.0 mg/kg IV) and ketamine (0.5 mg/kg IV) and maintained on a mix of isoflurane and oxygen. The dog received cefazolin (22.0 mg/kg IV) every 90 min intra-operatively and IV fluids (LRS at 5.0 ml/kg/h), as well as metoclopramide (0.3 mg/kg IV once), maropitant (1 mg/kg IV once), and a hetastarch bolus (4.9 ml/kg once). Constant rate infusions (CRIs) were administered intra-operatively for pain control (ketamine 10 mcg/kg/min and lidocaine 30 mcg/kg/min). Enbloc excision of the mediatinal mass was performed *via* median sternotomy using a bipolar vessel sealing device (LigaSure; Covidien, Mansfield, MA). A partial lung lobectomy of the cranial right lung lobe using a thoracoabdominal 30 mm vascular stapling cartridge was performed due to an adhesion to the mediastinal mass. Initially there was leakage of air from the partial lung lobectomy staple line which was resolved by oversewing with 5-0 Prolene. The mediastinal mass and sternal lymph nodes were submitted for histopathology. A 14 g MILA chest tube was placed. No complications were encountered in the perioperative period and the dog recovered uneventfully. The dog received ropivacaine (1.9 mg/kg) infiltration as an incisional block and carprofen (2.2 mg/kg) subcutaneously in recovery.

Post-operatively, the dog was monitored in the Intensive Care Unit. The dog was managed on IV fentanyl (2-4 mcg/kg/h) for the first day post-operatively then transitioned to methadone (0.1 mg/kg IV every 6 h as needed), IV plasma-lyte (55.5 ml/kg/day), trazodone (4.8 mg/kg PO every 8 h as needed), gabapentin (9.6 mg/kg PO every 8 h), carprofen (1.6 mg/kg PO every 12 h) and amoxicillin and clavulanic acid (16.0 mg/kg PO every 12 h) PO to address the suspected anal sacculitis. The chest tube was aspirated and fluid/air production was quantified every 8 h, and was subsequently pulled 48 h post-operatively. The dog was discharged from the hospital 72 h post-operatively.

**Figure 2:**
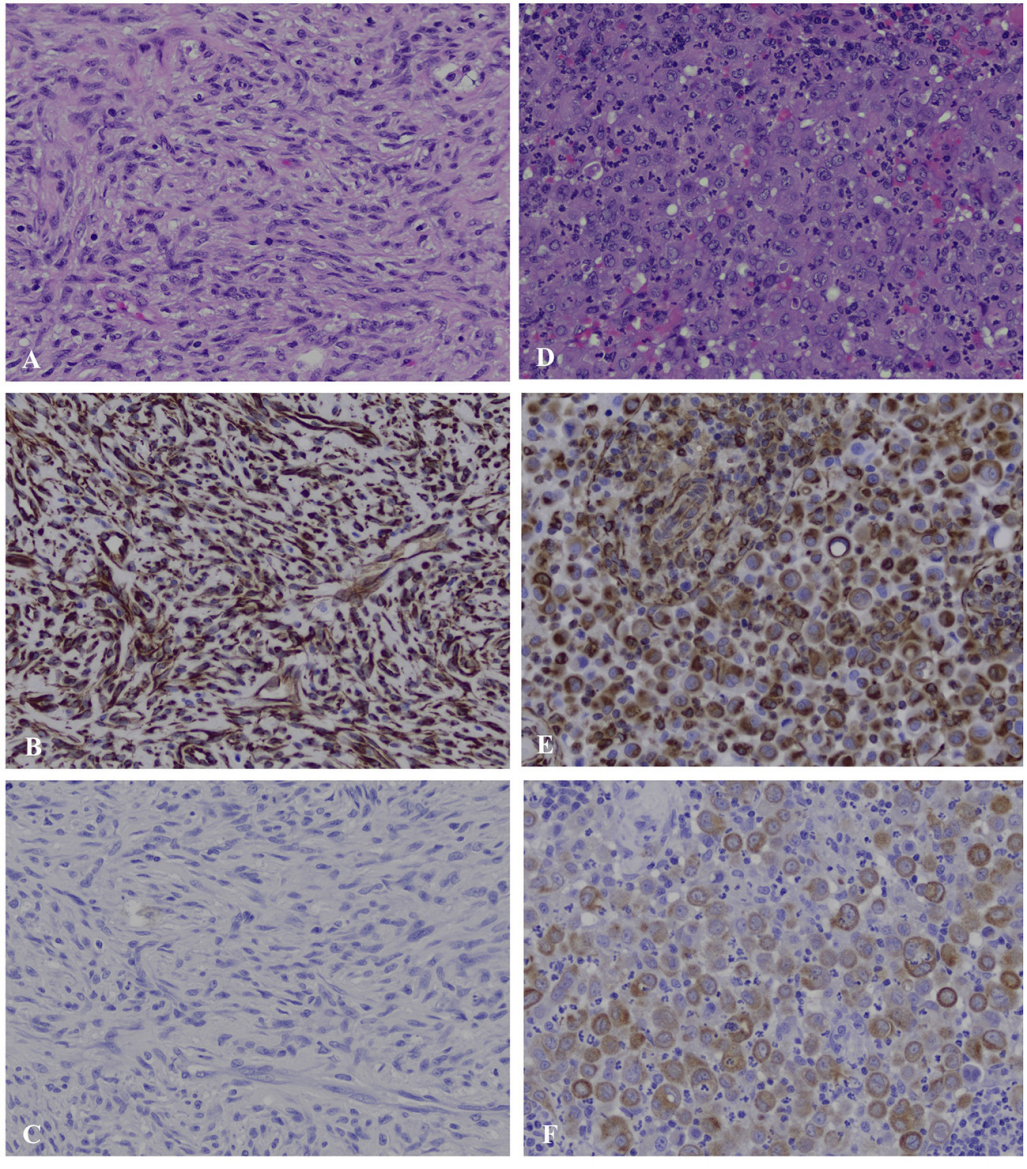
Mediastinal mass **(A–C)** and sternal lymph node **(D–F)**, canine. **(A)** The mediastinal mass is composed of densely packed, disorganized, interwoven bundles and streams of neoplastic spindle cells on scant fibrous stroma (40X). **(B)** Immunohistochemistry for vimentin of the mediastinal mass shows strong intracytoplasmic labeling within neoplastic spindle cells. **(C)** Immunohistochemistry for cytokeratin of the mediastinal mass shows no labeling among the neoplastic spindle cells. **(D)** The subcapsular and medullary sinuses of the sternal lymph node are infiltrated by sheets of pleomorphic round cells exhibiting marked anisocytosis and anisokaryosis, frequent binucleation in which the nuclei are squished together creating a flattened appearance of their coalescing borders, and emperiopolesis. **(E)** Immunohistochemistry for vimentin of the sternal lymph node shows strong intracytoplasmic labeling within pleomorphic round cells. **(F)** Immunohistochemistry for cytokeratin of the sternal lymph node shows moderate to strong intracytoplasmic labeling within pleomorphic round cells.

Histopathology of the mediastinal mass and associated lung tissue revealed disorganized interwoven bundles and streams of neoplastic spindle cells on scant fibrovascular stroma with 42 mitoses per ten 400X fields most consistent with fibrosarcoma (Figure 2). There was 10 mm of non-infiltrated lung tissue separating neoplastic tissue from the surgical margin reflective of complete excision. Histopathology of the sternal lymph nodes revealed sheets of pleomorphic round cells expanding the subcapsular and medullary sinuses. These cells exhibited marked anisocytosis and anisokaryosis, frequent binucleation, and emperipolesis. Based on these features, moderate mitotic activity (10 mitoses per ten 400X fields), and lack of morphologic similarity to the mediastinal mass, the presence of a separate neoplastic process within the lymph node was considered. Differential diagnoses for the sternal lymph node included poorly differentiated round cell neoplasm, carcinoma, and mesothelioma. Immunohistochemistry (IHC) was performed for both the mediastinal mass and sternal lymph node. The mediastinal mass was positive for vimentin and negative for cytokeratin AE1/AE3, supporting the initial diagnosis of fibrosarcoma by H and E staining. The atypical cells within the lymph node were interpreted as reactive mesothelial cells draining to the regional lymph node based on immunolabeling for both cytokeratin AE1/AE3 and vimentin, as well as their characteristic binucleated appearance in which the nuclei are squished together resulting in a flattened appearance of their coalescing borders (Figure 2). Given these findings, it was recommended to continue follow-up with the medical oncology service at the University of Illinois.

Approximately 1 month following surgery, the dog was presented for the first medical oncology follow-up appointment and cytotoxic chemotherapy was recommended. Thoracic radiographs were performed to restage and revealed no evidence of pulmonary metastasis though pleural fissures between the right lung lobes were noted and suspected to be either due to a small amount of pleural effusion or pleural thickening. The dog was started on doxorubicin (30 mg/m^2^) administered IV every 3 weeks with plan for a total of five to six treatments. Diphenhydramine (1 mg/kg IM) was administered before each doxorubicin treatment. A complete blood count, serum biochemistry and urinalysis were also performed at this visit. The complete blood count was unremarkable [HCT 57.8% (RI: 35.0–52.0%), WBC 6.89K/μl (RI: 6.0–17.0K/μl), neutrophils 5.44K/μl (RI: 3.0–11.5K/μl), PLT 239K/μl (RI: 200.0–700.0K/μl)], on the serum biochemistry there was a mild decrease in phosphorus level [1.9 mg/dl (RI: 2.7–5.2 mg/dl)] and urinalysis was unremarkable. A complete blood count was performed before every treatment to assess for myelosuppression and determine if chemotherapy could be continued. Thoracic radiographs were repeated following three doses of doxorubicin, the results revealed static mild pleural effusion with no signs of metastasis. Maropitant, metronidazole and trazodone were prescribed on an as-needed basis. The dog continued to do well clinically and five doxorubicin treatments were completed 127 days following initial diagnosis.

At 155 days following initial diagnosis, the dog presented to the Medical Oncology service for a recheck due to intermittent coughing. While his physical examination was unremarkable at that time, thoracic radiographs revealed mildly progressive pleural effusion. Cytology of the pleural fluid was consistent with modified transudate (nucleated cell count: 4,770 cells/ μl, red blood cell (RBC) count: <10,000 cells/ μl, total protein: 3.4 g/dl, specific gravity: 1.023 and 100-cell differential count consistent with 32% macrophages, 50% nondegenerate neutrophils and 18% small lymphocytes) with no infectious organisms nor neoplastic cells identified. The dog returned 5 days later (at 161 days following initial diagnosis) for a thoracic and abdominal CT scan. The CT scan was performed under sedation (0.2 mg/kg butorphanol IV and 4.0 mcg/kg dexmedetomidine IV) with pre- and post-contrast imaging using 60 ml (1 ml/kg) of iohexol administered IV through a peripheral indwelling catheter. Results revealed multiple nodules affecting the thoracic wall, pleura, mediastinum and potentially the cranial mediastinal lymph nodes. The lesions were sampled for cytologic review though were non-diagnostic. Given the suspicion for recurrence and the multifocal nature of the dog's disease, it was recommended to initiate a tyrosine kinase inhibitor (Palladia at 2.5 mg/kg Monday, Wednesday, Friday) with the goal of inhibiting blood vessel formation, as well as proceed with radiation therapy (RT) to slow the progression of disease. The dog returned 10 days later to initiate RT. Physical examination revealed a new firm mass on the ventral thorax and muffled bronchovesicular sounds were ausculted ventrally. A gated CT scan was performed under general anesthesia for RT planning. The dog received his first and second RT treatments 5 days and 8 days later, respectively. The initial planned protocol was to administer 6 gy x 6 over 4 weeks to address the bulky disease in the chest and pleural space though only two fractions were administered (one targeting the cluster of lesions in the cranial chest and one targeting the caudal chest) due to intolerance. To date, the dog has survived 223 days since diagnosis.

## Discussion

In dogs, cranial mediastinal masses are commonly diagnosed. Differentials include thymoma, lymphoma and less frequently, ectopic thyroid carcinoma, carotid body tumors, and brachial cysts (9). Other rare diagnoses include cranial mediastinal carcinoma, such as neuroendocrine and anaplastic (5), as well as mediastinal parathyroid adenocarcinoma (10). There are few reported cases of cranial mediastinal sarcomas in dogs (11, 12). Based on the combination of the above findings for the current case report, the final diagnosis was fibrosarcoma of the mediastinum. To the authors' knowledge, this report represents the first case of fibrosarcoma primarily arising in the cranial mediastinum in a dog.

Cytology of the mass revealed the presence of atypical mesenchymal cells. Cytology obtained from soft tissue sarcomas (STS) can be non-diagnostic due to poor exfoliation of cells or presence of necrosis resulting in a reported 63 to 97% accuracy in diagnosis (13–15). Given this, biopsy is recommended to obtain a definitive diagnosis, as well as histological grade (13). In this case, histopathology was most consistent with a fibrosarcoma for the mediastinal mass. Immunohistochemistry was performed to differentiate fibrosarcoma from spindle-cell variant of type A thymoma. The positive intracytoplasmic labeling of neoplastic spindle cells for vimentin and negative labeling for cytokeratin was consistent with their mesenchymal origin and supportive of the diagnosis of fibrosarcoma (Figure 2). The pleomorphic round cells within the sternal lymph node exhibited strong intracytoplasmic labeling for both vimentin and cytokeratin supporting the diagnosis of reactive mesothelial cells (Figure 2). There are reports in human literature of hyperplastic mesothelial cells within the sinuses of lymph nodes of patients presenting with pericardial, pleural, or abdominal effusions secondary to lymphoproliferative disease, congestive heart failure, and inflammatory diseases (16–18). It is postulated that the presence of effusions leads to disruption of mesothelial cells lining the pleura allowing them to drain into subpleural lymphatics (16). Ultimately, in these cases, a definitive distinction between benign vs. malignant mesothelioma cannot always be made, as benign and malignant mesothelial cells often share many of the same histologic features. One paper reported the use of desmin and epithelial membrane antigen (EMA) as IHC markers to distinguish malignant from reactive/benign mesothelial cells in human cytologic effusions (19). Although desmin has been validated in dogs for IHC, it remains yet to be determined whether EMA is a valid marker in canine tissues. Regardless, clinical follow up revealing lack of effusions or tumor recurrence would suggest a benign process.

Fibrosarcomas (FSAs) arise from malignant fibroblasts and can occur in any location though most are found in the skin, subcutaneous tissue and the oral cavity of older dogs and cats with no breed or sex predilection (13). FSAs range from well-differentiated to anaplastic (13, 20). When compared to other subtypes of STS, they are more likely to recur after incomplete excision and have higher mitotic rates though are more likely to be low grade (14, 21–23). Factors that increase the risk of metastasis, most commonly to the lungs, include histologic grade, number of mitotic figures, percentage of tumor necrosis and local tumor recurrence with an overall metastatic rate of 0% to 31% (13, 14, 21, 23–28).

While fibrosarcoma has not been previously well-described within the cranial mediastinum of a dog outside of a larger retrospective, observational cross-sectional study aimed at assessing radiographic differentiation of mediastinal vs. pulmonary masses (29), when STS are isolated to other locations, local tumor control is the primary goal of treatment. This is usually accomplished with surgical resection that can be sometimes followed by adjuvant radiation therapy. Radiation therapy, most commonly palliative, has also been used as a single modality (13). The median survival time for dogs with STS (excluding those arising from the oral cavity) following surgery alone ranges from 1,013 to 1,796 days and increases to 2,270 days with surgery and adjunctive RT (13, 22, 23, 30, 31). The role of chemotherapy in the management of dogs with STS is controversial though may be beneficial in decreasing the rate of local recurrence (13). However, survival is dependent on feasibility of local tumor control which can be challenging when localized to the mediastinum. In a recent retrospective case series of miniature schnauzers diagnosed with intrathoracic histiocytic sarcoma in which either surgery followed by Lomustine vs. Lomustine alone was pursued, the disease course was aggressive with short survival times suspected to be partly due to lack of feasibility of local treatment (11). Similarly, in a case of a primary cranial mediastinal hemangiosarcoma in a young dog, survival time was short (8 months) following surgical excision though chemotherapy was not pursued in this case (12).

In the human medical literature, mesenchymal tumors of the mediastinum are considered uncommon, in addition to primary thymic carcinomas, neuroendocrine carcinomas, germ-cell tumors, lymphomas and neurogenic and endocrine tumors, these comprise of <10% of all mediastinal masses (32). More specifically, fibrosarcomas, such as mediastinal fibromatosis, are rare tumors of the anterior mediastinum that are locally invasive but rarely metastasize. Diagnosis is often one of exclusion and requires histopathology. Often despite surgical resection, chemotherapy, both in neo-adjuvant and adjuvant setting, and postoperative radiation therapy if surgical margins are incomplete, the prognosis remains poor. Larger masses often reoccur, whereas the smaller lesions have a higher likelihood of metastasizing (32, 33).

In this case, the mass was completely excised surgically, and the dog was prescribed systemic cytotoxic chemotherapy with doxorubicin, an anthracycline antitumor antibiotic. Maximum tolerated dose (MTD) chemotherapy is often recommended in STS cases that have evidence of metastasis at time of diagnosis or a high likelihood of metastasizing. There is no current evidence to suggest a survival advantage for dogs; however, doxorubicin or ifosfamide are the agents of choice based off human oncological practice (13). In a meta-analysis of 18 human studies evaluating the use of adjuvant chemotherapy for localized resectable STS of all grades, adjuvant doxorubicin, with or without ifosfamide, was found to provide modest benefits in local tumor control, metastatic control, and overall survival amongst nearly 2,000 patients (34). In comparison, a previous study evaluating treatment of various canine neoplasms using doxorubicin found that a two-dose treatment resulted in at least a 50% volume reduction in one of 14 dogs affected by fibrosarcoma (35).

In conclusion, primary cranial mediastinal fibrosarcoma, although a seemingly rare cause of thoracic pathology in dogs, should be considered in the differential diagnosis for a cranial mediastinal mass. Surgical excision and adjuvant doxorubicin chemotherapy were performed in this case for local control. Recurrence was noted at 161 days following diagnosis and Palladia and stereotactic radiation therapy were added to the dog's treatment regimen.

## Data Availability Statement

The original contributions presented in the study are included in the article/supplementary material, further inquiries can be directed to the corresponding author.

## Ethics Statement

Ethical review and approval was not required for the animal study because only case report. Written informed consent for participation was not obtained from the owners because only case report.

## Author Contributions

AM wrote the manuscript. LS assisted in supervision of the surgical management of this case and contributed to conception of the case report. SS supervised the surgical management of this case. AS supervised the clinical management of this case with assistance from AL. JL and ML assisted in pathology reviewing. All authors critically reviewed and approved the final version of the manuscript.

## Conflict of Interest

The authors declare that the research was conducted in the absence of any commercial or financial relationships that could be construed as a potential conflict of interest.

## Publisher's Note

All claims expressed in this article are solely those of the authors and do not necessarily represent those of their affiliated organizations, or those of the publisher, the editors and the reviewers. Any product that may be evaluated in this article, or claim that may be made by its manufacturer, is not guaranteed or endorsed by the publisher.
